# Design of gas control lane of 9# coal seam in Wuhushan Mine based on layer layout optimization

**DOI:** 10.1038/s41598-024-77020-6

**Published:** 2024-10-26

**Authors:** Chun Zhang, Xianju Qian

**Affiliations:** 1 College of Safety Science and Engineering, Liaoning Technical University, Fuxin, 123000 Liaoning China; 2 Key Laboratory of Mine Thermodynamic Disasters and Control of Ministry of Education, Liaoning Technical University, Huludao, 125105 Liaoning China

**Keywords:** Gas control lane, Upper corner, Fissure zone, Air extraction zone, Numerical simulation, Energy harvesting, Energy storage

## Abstract

In order to address the issue of gas over limit in the upper corner of the working face of the 9# coal seam in Wuhushan Mine, a series of theoretical and numerical simulation analyses were conducted to evaluate the optimal configuration for the gas control lane of the 9# coal seam. In accordance with the "O" circle theory and the lithology of the overlying rock strata of the 9# coal seam, the height range of the fallout zone and fissure zone in the working face mining area was determined by employing empirical formulas. The change rule and distribution characteristics of the porosity of the fissure zone and the fall zone in the mining area were analyzed based on the characteristics of rock movement and fall. The determination method was also provided. The numerical simulation software was employed to simulate and analyze the gas concentration field in the air-mining zone under conditions of no extraction and six distinct layer positions of the gas control lane. The optimal layer position of the gas control lane in the 9# coal seam was determined and subsequently implemented in the field. The results demonstrate that the overlying rock layer in the 9# coal seam exhibits a height range of 6.86 ~ 11.26 m, while the fissure zone displays a height range of 30.11 ~ 41.31 m. When the gas control road is situated in close proximity to the working face, the gas concentration field exhibits a markedly low concentration. When the distance between the gas control lane and the return airway of the working face is 20 m and the distance from the top of the coal seam is 20 m, the gas concentration in the upper corner and the return airway is 0.35% and 0.26%, respectively. These values are close to the lowest concentration observed in the layout scheme. Additionally, the gas extraction concentration and the pure volume of the gas control lane are 23.7% and 38.3 m^3^ min^−1^, respectively. These values represent the highest concentrations observed in the various layout schemes. The application of the gas management lane in the field, based on the numerical simulation results, demonstrated a successful extraction effect, which was consistent with the numerical simulation results. This effectively managed the issue of an over-limit of gas in the upper corner of the working face of the 9# coal seam.

## Introduction

As a consequence of the accelerated growth of China’s economy, the demand for energy is rising, and coal^1–4^, which constitutes the primary source of energy in China, is becoming increasingly significant. However, the issue of gas disasters in the context of coal mining is becoming increasingly prevalent. It represents a significant challenge to coal mine safety and production. There is an observable increase in the amount of gas gushing out of the mine^5,6^, as well as an expansion in the gas limit at the upper corner of the working face^7,8^. This phenomenon poses a considerable threat to the miners’ lives and the safe operation of the mines^9,10^. In recent years, the occurrence of gas explosion accidents has resulted in significant losses for the country, mining enterprises, and miners alike^6^.

The deformation and destruction law^11^ and gas transportation law^12,13^ of the strata in the mining process have been the subject of extensive study by scholars for many years. In accordance with the "O" circle theory^14,15^, as the working face progresses, the overlying rock layer of the mining area undergoes collapse, and the fissures of the rock layer in the center of the roof plate are compacted. Conversely, some fissures around the mining area are retained due to the supporting effect of the coal wall. As a result, the mining area and the surrounding rocks experience varying degrees of stress changes^16,17^, top and bottom plate deformation^18^, and fissure evolution in accordance with the aforementioned phenomena. Sun et al.^19^ investigated the deformation and migration patterns of the overburden in deep and extra-thick coal seam mining, employing comprehensive monitoring techniques and empirical formulas. The research team devised a novel method for monitoring the deformation and damage of the overburden through distributed fiber-optic sensing technology, which offers a pioneering experimental approach to averting and managing mine disasters. Qin et al.^20^ conducted research into the transport law of the overlying rock layer in the deep hollow zone of Pinggang Mine. They designed a technology system, designated the "transport-transfer-control" system, which was to be used for the peripheral rock in the working face of the hollow zone. Additionally, they put forward three kinds of tunnel support programs, which provided theoretical and technical support for controlling the deformation of the roadway. In their study, Lv et al.^21^ employed a combination of theoretical calculations and numerical simulation methods to investigate the deformation process of the coal and rock seams in the air-mining zone, the stress changes in the overlying and underlying coal seams, and the mode of transportation of gas in the protective layer after depressurization. Their findings confirmed the location of the gas aggregation zones, which they combined with the geologic conditions of the study area. A comprehensive examination of the research findings allows for a thorough comprehension of the stratum deformation and destruction mechanisms, as well as the gas flow law governing the mining process in air-mining zones.

The implementation of a gas management lane^22–24^ represents a viable approach for effectively addressing elevated gas concentrations. The judicious deployment of coal seam gas management lanes can enhance the efficiency of gas extraction, curtail the volume of gas outflow, and mitigate the risk of gas accidents. In the present era, the method of evaluating and optimizing the gas extraction process through computer numerical simulation^25–27^ is widely utilized. Bing et al.^28^ concentrated their efforts on the numerical simulation based on FLAC3D software to determine the radius of gas extraction, which was subsequently applied to the No. 3 coal seam of the Changcun coal mine and yielded favorable outcomes. Zhao et al.^29^ and Liu et al.^26^ employed numerical simulation techniques to ascertain optimal drilling sealing depths and to optimize the parameters of gas drilling in the extraction zone. Ultimately, they were able to confirm the optimal location of extraction drilling holes and observed an enhanced concentration of extracted gas in the field application. Zhou et al.^30^ employed COMSOL numerical simulation and field testing to optimize two parameters: the spacing of pipe inserts and the negative pressure of mining. This was done with the objective of determining the optimal location of drilling holes and spacing of pipe inserts. The resulting optimization effectively improved the amount of gas gushing out. It is evident that scholars have conducted comprehensive research on the configuration of the gas control lane, the layout mode, and the determination of extraction parameters, among other aspects. The findings of these studies offer a scientific foundation and theoretical guidance for the management of gas in the upper corner of the airspace zone.

While the aforementioned studies have yielded substantial insights through diverse methodologies, including theoretical analysis, numerical simulation, and field experimentation, they have nevertheless encountered inherent limitations. As the numerical simulation of the air-mining zone is based on certain assumptions and simplifications of complex geological conditions, such as the setting of rock mechanical parameters, the dynamic flow characteristics of the gas, the boundary conditions of the model, and other parameters, it may not be possible to comprehensively represent the complex geological conditions of the coal mines under study. Furthermore, the theoretical analysis may not be able to cover all the factors affecting the layout of gas management lanes, which may ultimately result in a significant discrepancy between the simulation results and the actual situation. It is possible that the final simulation results may deviate significantly from the actual situation. Accordingly, the subsequent study must establish a more sophisticated model and consider a multitude of variables to enhance the precision of the ultimate simulation outcomes. Concurrently, a comparative analysis of multiple coal mines with analogous geological characteristics is undertaken to substantiate the generalizability of the findings of this study.

This paper presents an analysis of the gas concentration in the upper corner, the gas concentration in the return-airway, the gas concentration in the roadway, and the pure amount of gas extraction. The analysis reflects the influence of the layered position of the gas control lane on the effect of gas overrun control from different perspectives and determines the optimal position of the gas control lane in the 9#. The coal seam is more accurately delineated, thereby enhancing the efficiency of gas extraction in the high-efficiency pumping roadway. This ensures the safety and efficiency of production in the mine, increases the economic efficiency of the enterprise, and contributes to the government’s "double-carbon" policy^31^.

## Analysis of layered positions and determination of relevant parameters

### Engineering background

**Fig. 1:**
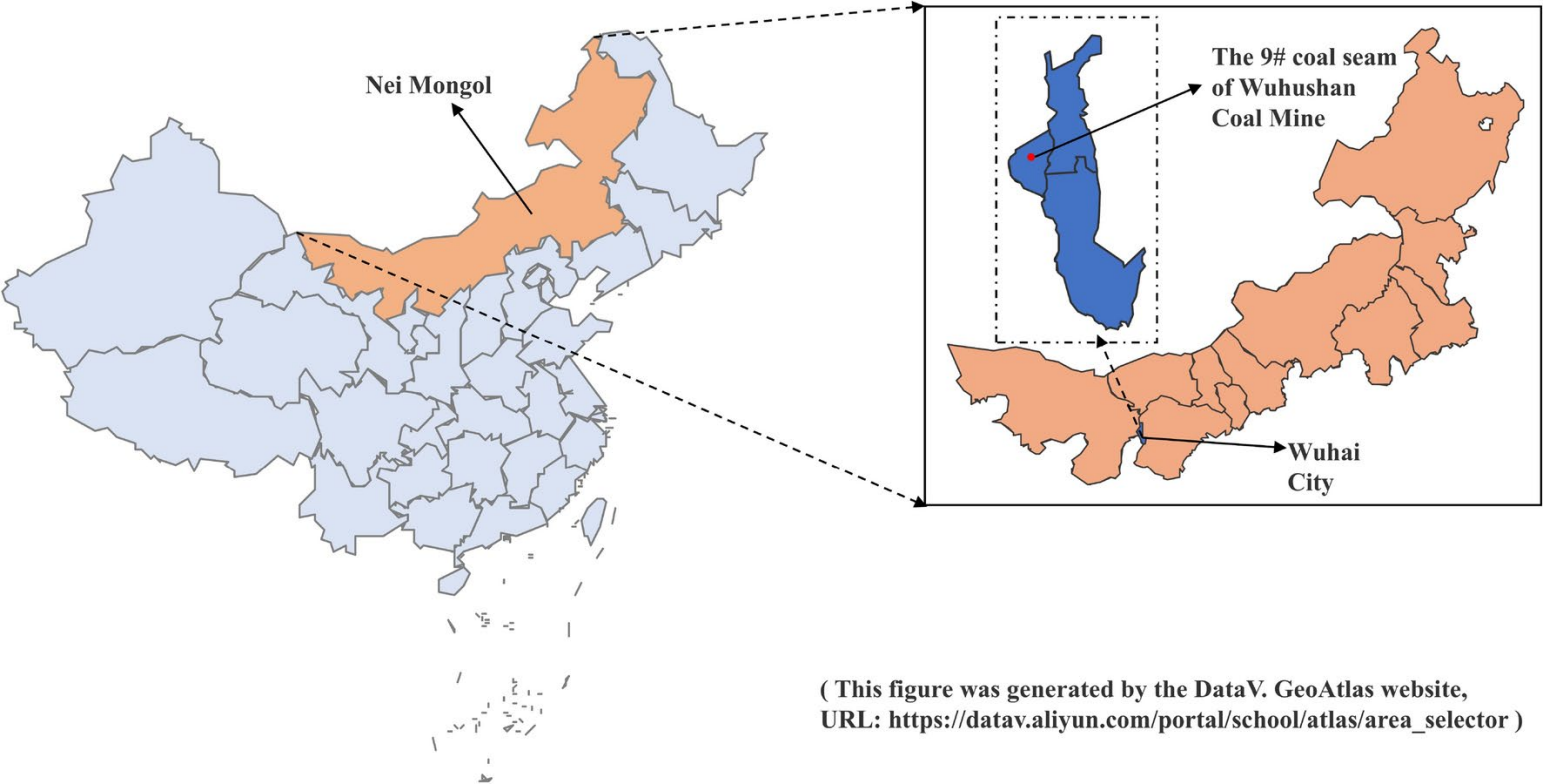
Geographical location map of the 9 # coal seam in Wuhushan, China.

The Wuhushan mine is situated in Wuhai City, Inner Mongolia Autonomous Region, China. It is under the ownership of Shenhua Group Wuda Wuhushan Limited Liability Company, the location of which is indicated in Fig. 1. The mine is situated in the southwestern sector of the Wuda basin, which exhibits asymmetric tilt. The folds within the well field are relatively gentle, indicative of an overall monoclinic tectonic trend. The direction of this tectonic movement can be approximated as north–south, with an eastward inclination. The strata inclination angle ranges from 5° to 15°, and the well field spans a north–south length of approximately 7 km. The well field has an east–west width of 3.9 km and a length of approximately 29.25 km^2^. It is situated at an east–west distance of 5 km from the center of the mine. The coal-bearing strata in the well field span the Carboniferous to the Permian periods and comprise a total of eight layers of recoverable coal seams. The main recoverable coal seams are 9#, 10#, and 12#.

According to the official survey report of the Wuhushan Coal Mine, the average thickness of the 9# coal seam in the Wuhushan Mine is 3 m. The structure of the coal seam is complex, comprising 2 ~ 4 coal seams and 1 ~ 3 layers of interbedded rocks. The working face has a length of 200 m in the direction of arrangement, an advanceable length of 1388 m, and a mining area of approximately 208,552 m^2^, with a mineable reserve of 818,000 t and a geologic reserve of 911,000 t. Based on the calculation of 2800 t per day, the predicted mining of the seam is expected to result in a relative gas outflow of 14.43 m^3 ^t^−1^ at the working face, with an absolute outflow of 53.89 m^3^ t^−1^. The top plate of the 9# coal seam is primarily composed of sandstone, including granular sandstone, fine sandstone, siltstone, and coarse sandstone. The specific structure and thickness of the top plate rock layer are presented in Table 1.

**Table 1:**
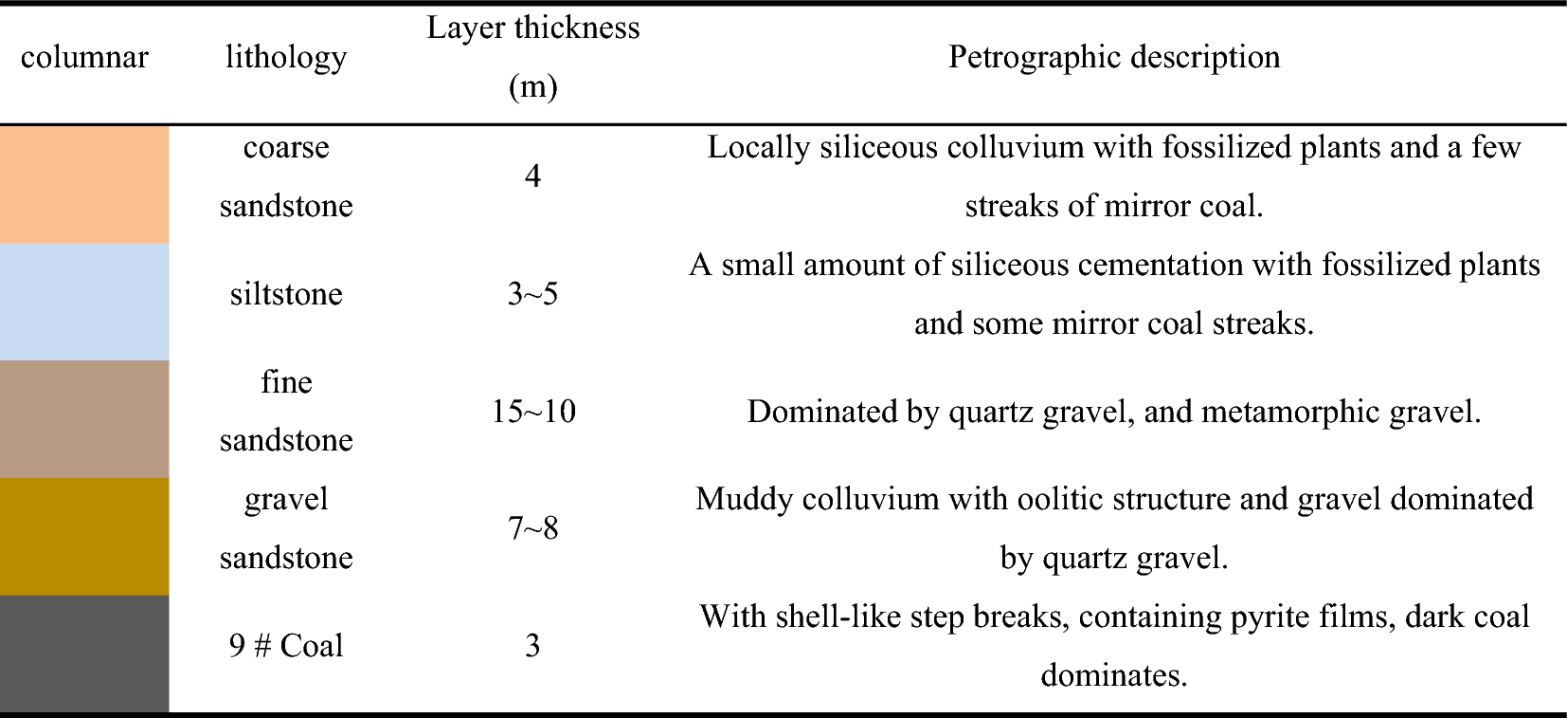
Characteristics of rock stratum at the top of 9# coal seam.

### Layout layer analysis

To determine the optimal location for the gas control lane, it is necessary to consider two key aspects. Firstly, the gas control lane should be situated as close as possible to the area of high gas concentration in the mining area, which is typically conducive to gathering gas and has a higher extraction efficiency. Secondly, the fissures where the gas control lane is located should be developed as far as possible to increase the permeability of the rock layer and enhance the influence range of the gas control lane^32^. This paper also primarily examines these two factors when determining the layout location of the gas control lane for the 9# coal seam. In this paper, the determination of the layer position of the 9# coal seam is primarily based on these two points.

Based on a synthesis of prior theoretical research and field experience, the gas control lane is typically situated within the fissure zone above the fallout zone of the mining area. The fissure of the rock layer in this area is more developed, with better permeability, and the gas control lane is relatively intact after the fallout zone of the rock layer. This allows the gas control lane to continue to satisfy the needs of gas extraction in the mining area^33,34^. Additionally, the gas control lane is not easily accessible from the mining area, which helps to maintain the concentration of gas extraction and prevent the risk of increasing the leakage of air in the mining area. The potential for air leakage in the mining area is heightened.

#### Determination of the location of vertically oriented gas control lanes

In general, the closer the fissure zone is to the fallout zone, the more favorable the permeability of the rock layer after movement and deformation, which in turn makes the gas control lane more effective in regulating gas in the mining zone. However, due to the considerable dispersion of the mechanical properties of the rock body^35^, when the gas control lane is laid close to the fallout zone in actual production if the rock layer on the top plate of the mining area is characterized by a high-risk zone, it is prone to collapse, which reduces the extraction effect and may even result in spontaneous combustion of the coal in the mining area.

Accordingly, when installing the gas control lane, a specific safety distance must be maintained from the upper limit of the fallout zone. The requisite safety distance should be determined based on the specific circumstances of the site. The fundamental principle governing the determination of the safety distance for the gas control lane at the 9 #working face is as follows: It is essential to ensure that, in the event of effective management of the over-limit of gas at the upper corner of the working face, the drooping distance (the distance from the top of the coal bed) of the gas control lane is as far as possible, to guarantee the effectiveness of gas management at the upper corner and, consequently, the effectiveness of gas management at the upper corner. This approach ensures the efficacy of gas control in the upper corner and guarantees the long-term and efficient application of the gas control tunnel.

#### Determination of the location of horizontally oriented gas control lanes

The location of the gas control lane in the horizontal direction is typically situated within the incorrect working face back-air lane for a certain distance. The optimal distance should be determined based on the specific circumstances. A shorter distance may be advantageous for managing gas over limits in the upper corner of the working face. However, it may result in increased leakage of air from the working face back-air lane to the gas control lane, leading to a reduction in the concentration of extracted gas and the efficiency of gas extraction in the mining airspace. Furthermore, the influence of coal columns on the side of the back-air lane results in a deterioration of fissure development in the fissure zone of the rock overlying the roof plate, with proximity to the gas flow and storage being a contributing factor. Furthermore, the influence of the coal column in the side section of the return-air lane results in a deterioration of fissure development in the fissure zone of the overlying rock layer on the roof plate as the distance to the return-air lane decreases. This, in turn, creates an unfavorable environment for the flow and storage of gas.

Accordingly, the distance between the gas control lane and the return airway in the horizontal direction should be neither excessive nor insufficient^36^. Based on the theoretical research and practical production experience of researchers and scholars^33^, the optimal range for this distance is generally within the range of 10–30 m. In this study, we analyze the specific location of the horizontal direction of the gas control lane of the 9# coal seam of the Wuhushan Mine within this scope.

### Determination of the height of the “two zones” of the overlying rock formation

With the advancement of the working face, the deformation, migration, and collapse^37^ of the overlying rock layers in the goaf result in the formation of caving zones, fracture zones, and curved subsidence zones from bottom to top in the vertical direction of the goaf. The caving zones and fracture zones are referred to as the “two zones” for short. Many factors affect the height of the “two belts”, including the depth of the goaf, the thickness of the coal mining, the properties of the rock layers, and the mining speed^38^. The determination of the height of the caving zone and fracture zone in the overlying rock layer is the basis for the reasonable layout of gas control tunnels. Based on the lithology of the coal seam roof, the height of the caving zone and fracture zone in the goaf roof can be determined according to empirical formulas.

The height of the fallout zone is typically three to five times that of the mining height. This area is essentially formed following the collapse of the direct top rock layer subsequent to the mining of the working face. According to the actual engineering situation, the lithology of the overlying rock of the coal seam roof is divided into four categories based on the uniaxial compressive strength of the rock: hard, medium hard, weak, and extremely weak^39,40^. The strength classification of the “two belts” overlying rock is shown in the Table 2. According to the lithology of the roof, it is known that the rock layer in the upper caving zone of the 9 # coal seam is sandstone, which belongs to the medium hard rock layer. The empirical formula for calculating the height of the fallout zone is provided in Eq. (1) ^41^.

1 $$H_{C} = \frac{100\sum M }{{4.7\sum M + 19}} \pm 2.2$$

where *H*_*C*_ is the height of the fallout zone, m; ∑*M* is the cumulative height of the coal seam mined, m. The thickness of the 9# coal seam is 3 m, and it can be calculated that the height of the fallout zone in the mining zone is about 6.86 ~ 11.26 m.

**Table 2:** The strength classification of the “two belts” overlying rock is shown in the table.

Lithology of Overburden Rock	Uniaxial compressive strength range/MPa	Representative rocks
hard	[40, 80)	Quartz sandstone, limestone, conglomerate
Medium hard	[20, 40)	Sandstone, mudstone, sandy shale, shale
weak	[10, 20)	Mudstone, mudstone sandstone, bauxite, weathered mudstone
Extremely weak	< 10	Clay, sandy clay

The fissure zone is situated above the fallout zone, and a considerable number of secondary fissures are formed following the movement of the rock layer, providing channels and space for the transportation and storage of gas. The lithology of the roof slab indicates that the rock layer of the fissure zone in the overlying 9# coal seam is also a medium-hard rock layer. The empirical formula for calculating the height of the fissure zone is presented in Eq. (2) ^32^.

2 $$H_{L} = \frac{100\sum M }{{1.6\sum M + 3.6}} \pm 5.6$$

where *H*_*L*_ is the height of the fissure zone, m. According to the thickness of the 9# coal seam is 3.0 m, the height of the fissure zone can be calculated to be about 30.11 ~ 41.31 m. The height of the fissure zone can be calculated to be about 30.11 ~ 41.31 m. Analysis suggests that the vertical distance of gas control tunnels should be between the collapse zone and the fracture zone^33^. Namely 11.26 ~ 30.11 m.

Above, based on the literature and empirical formulas, the horizontal distance of the gas control lane is estimated to be roughly within the range of 10–30 m, but the analysis based on numerical simulations and theoretical calculations shows that the optimal horizontal distance of the gas control lane should be in the range of 15–30 m under specific geological and engineering conditions. In the range between the fallout zone and the fissure zone is usually located at or near the direct roof of the coal seam, where the movement and stress distribution of the overlying rock layer is relatively stable, and neither collapses as in the case of the direct roof, nor is the rock layer too hard to form an effective fissure. And in this range the porosity is higher, the gas flow resistance is smaller, which is favorable for the transportation of gas, and it can be more easily propagated through these fissures to the management lane, and a certain safety coefficient is taken into account. Therefore, the reasonable layout of the gas management lane in the 9# coal seam’s air-sea mining area, the horizontal distance is between 15 ~ 30 m, and the vertical distance should be between 12 ~ 31 m.

### Analysis of the distribution characteristics of porosity in Goaf

The void fraction distribution characteristics of the mining area exert a profound influence on the transportation and storage of gas and are also the pivotal parameters when simulating and analyzing the optimal layering of the gas control lane. Therefore, it is imperative to analyze the void fraction distribution characteristics of the mining area. As the gas control lane is situated within the fissure zone of the overlying rock layer in the air mining area, the porosity of the rock layer in the fissure zone and the distribution of the porosity of the rock fall in the air mining area exhibit significant discrepancies. Consequently, it is imperative to conduct a detailed analysis of the porosity of the rock layer in the fissure zone and the porosity of the rock fall in the air mining area as separate entities.

#### Characterization of void fraction distribution in the fallout zone

The change in the porosity of the fallout rock within the mining airspace is primarily influenced by the pressure exerted by the roof plate^42^. The regular polynomial that depicts the alteration in the porosity of the crushed rock body in response to stress along the advancing direction of the working face is illustrated in Eq. (3).

3 $$\alpha_{1} = \beta_{3} \sigma^{3} + \beta_{2} \sigma^{2} + \beta_{1} \sigma + \beta_{0}$$

where* α*_*1*_ is the porosity; *σ* is the relative compressive stress of any cross-section of the tendency of the hollow zone, i.e., the vertical stress of the roof plate, MPa; *β*_*i*=1,2,3_ is the regression coefficient; *β*_*0*_ is the porosity of the crushed rock before it is subjected to the stress in the direction of the advancement of the working face, i.e., the porosity behind the free emergence of the rock.

**Fig. 2:**
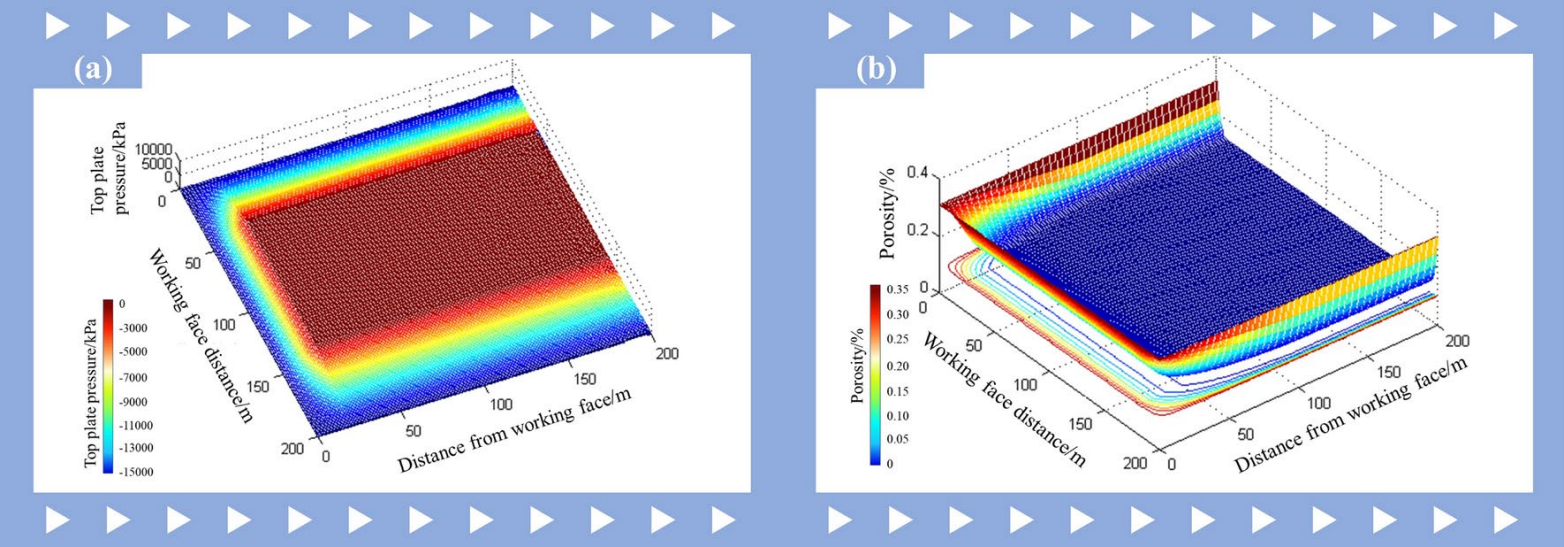
(**a**) the roof pressure distribution of goaf; (**b**) The change regulation of porosity.

By the "O" circle theory and considering the burial depth of the 9# coal seam, the cycle pressure step, the lithology of the roof plate, the section of the coal pillar, and other factors, the stress distribution of the 9# coal seam is illustrated in Fig. 2a. The stress near the working face and on both sides of the wind tunnel is relatively low, while the stress in the center of the working face is higher. Following the established principles governing the distribution of stress within the mining airspace, the porosity distribution of the fallout rock within this area was ultimately determined. This is illustrated in Fig. 2b.

As can be seen from the change regulation of porosity in Fig. 2b, the porosity varies in the direction of working face advancement (horizontal and vertical), and the porosity in different areas has a large difference. In the early stage of coal seam mining, the hollow area is formed, and the surrounding rocks will fall due to the loss of support. At this point, the porosity will begin to decrease as the rock is broken and displaced to fill most of the voids. As mining continues, the void zone expands, the fallout and fragmentation of the overlying rock continues, and the porosity continues to decrease. At this stage, the magnitude of porosity reduction mainly depends on factors such as the dip angle of the coal seam, rock structure and geological formation. When the mining area develops to a certain scale, the overlying rock layer will gradually fill in the gaps of the mining area, forming a dynamic balance, and the porosity will gradually decrease and tend to stabilize. At this stage, the porosity is mainly affected by the rearrangement and compression of the rock. Due to the influence of the support, the two sides of the mining area, the surrounding rocks are stretched and compressed during the sinking of the roof plate of the mining area, which leads to the further expansion of the cracks in the rocks, so the porosity of the two sides is larger than that of the middle position.

#### Distribution characteristics of porosity in fissure zone

In the direction of the working face advancement, the movement of the roof plate in the hollow area lags behind the advancement of the working face. This results in a discrepancy in the amount of movement and deformation of the rock layer in the fissure zone of the roof plate in the hollow area (i.e., the size of the void rate). In general, within a certain range of the roof, this region of the fissure zone exhibits minimal rock movement and a correspondingly low void rate. This can be considered the original void rate zone. Subsequently, within a certain distance of the inner extension of the air mining zone, the aforementioned region of the roof is subjected to a more pronounced influence from the mining activities of the working face. This results in a discernible increase in rock movement, the full development of rock fissures, and a notable expansion in the void rate. Consequently, this region can be designated as the void rate increases area. Subsequently, the movement of the rock layer ceases in the porosity increase area and the pressure exerted by the roof plate gradually intensifies. As a result of this pressure, the fissures in the rock layer gradually close, and the porosity gradually decreases. However, it remains larger than the original porosity of the rock layer, and this area can be designated as the porosity restoration area.

In the vertical direction, the porosity of the rock layer in the fissure zone of the extraction zone generally decreases gradually with increasing vertical distance, according to the negative exponential law. This trend continues until the porosity of the rock layer in the bending and sinking zone is almost unchanged.

In light of the preceding analysis, it is recommended that the porosity of the rock layer in the top plate fissure zone of the mining zone be determined on a separate basis for different areas. The porosity of the rock layer in the area where the original porosity remains unaltered by mining is largely unaffected by the mining process. The porosity in the area where the porosity increases and the porosity recovery area can be estimated by theoretical derivation using an Eq. (4).

4 $$\alpha_{1} = \left[ {H + \left( {1 - K_{p} } \right)H_{1} + \alpha \left( {H_{2} - H_{1} } \right)} \right],\left[ {H_{2} + H - K_{p} H_{1} } \right]$$

where* α*_1_ is the porosity; *α* is the original porosity of the rock layer in the fissure zone; *H* is the mining height of the working face, m; *H*_*1*_ is the height of the fallout zone, m; *H*_*2*_ is the height of the fissure zone, m; *K*_*p*_ is the coefficient of fracture of the rock in the fallout zone (porosity restoration area) or the coefficient of residual fracture (porosity increase area). According to the survey report of 9# coal seam of Wuhushan Mine and the test results of mechanical parameters of rocks^43^, the range of crushing coefficient of medium-hard rocks is 1.2 ~ 1.3. The values of each parameter in the Eq. (4) are shown in Table 3.

**Table 3:** Calculation parameters of porosity in the fracture zone of the mining area.

Original porosity	Mining height	Drop zone height	Fissure zone height	Coefficient of dilatancy	Residual coefficient of expansion
0.07	3m	11.26m	41.31m	1.2	1.03

By bringing the values in Table 2 into the Eq. (3), the porosity is calculated to be 14.6% in the porosity increase zone of the fissure zone and 9.3% in the porosity recovery zone.

## Numerical simulation analysis

### Relevant parameters and boundary conditions

#### Oxygen consumption rate of residual coal

The oxygen consumption rate of coal remaining in the mining area is primarily influenced by the oxygen concentration and reaction temperature^44,45^. The test analysis indicates that as the oxygen concentration increases, the oxygen consumption rate of coal accelerates in a linear fashion. Furthermore, as the reaction temperature rises, the oxygen consumption rate of coal increases exponentially, with a doubling of the rate occurring at approximately the same temperature^46,47^. The corresponding formula is shown in the Eq. (5).

5 $$W_{{\left( {{\text{O}}_{2} } \right)}} = \frac{{(1 - n)H_{1} \gamma_{0} c\left( {{\text{O}}_{2} } \right)}}{{nHc\left( {{\text{O}}_{2} } \right)_{0} }}{\text{e}}^{\phi t}$$

where *n* is the porosity, *c*(O_2_) is the oxygen concentration, mol/m^3^; *H* is the height of the air-mining zone, and *H*_1_ is the height of the relict coal stacking layer; *γ*_0_ is the oxygen consumption rate constant of the coal, which is a coefficient to be determined, mol/(m^3^·h), and the specific value of the literature content is taken here to be 1.472 × 10^−5^mol/(m^2^·s); *φ* is an experimental constant, here φ = 0.0235℃^−1^ is taken ^48^; *t* is the temperature of the mine area, ℃.

#### Gas outflow intensity

The source of gas outflow from the working face is primarily comprised of coal wall gas, fallen coal gas, coal residue gas in the mining area, and gas outflow from neighboring layers^49^. The influence of working face mining or coal mining machines’ coal remnants results in alterations to the original conditions of gas accumulation in the coal seam. This leads to an increase in the area of coal seam fissures and exposed air, which in turn causes part of the free gas to gush out from the coal wall or coal remnants. Consequently, the volume of gas emitted is contingent upon the duration of exposure of the coal body to the atmosphere and the quantity of residual coal.

According to the observation of field practice, the intensity of gas outflow from the coal remains in the mining area decreases with the extension of the exposure time of the coal remains according to the negative exponential law^50^, and the calculation formula is shown in Eq. (6).

6 $$Q_{{(CH_{4} )}} \left( {\text{X}} \right) = \varepsilon e^{{ - 1440\lambda \frac{X}{V}}}$$

In the formula: *Q*_*(CH4)*_(X) is the gas emission intensity at a distance of X m from the working face in the goaf, m^3^/(m^2^·min);* ε* is the maximum gas emission intensity when the residual coal in the goaf is just exposed, m^3^/(m^2^·min); Based on the absolute gas emission rate of the goaf of coal seam 9#, the maximum gas emission intensity at the time of initial exposure of residual coal is taken as 8.769 × 10^–7^ m^3^/(m^2^·min); *λ* is the attenuation coefficient of gas emission intensity, which is taken as 1.2 here; *V* is the advancing speed of the working face, m/d. Here, it is assumed that when the residual coal enters the goaf for 300 m, the gas emission is exhausted and the gas emission intensity is taken as 0.

#### Boundary conditions of seepage field

In the simulation when the working face is constantly leaking air into the mining airspace, the boundary conditions of the leakage seepage equation are shown in Eq. (7).

7 $$\left\{ {\begin{array}{*{20}c} {p|_{{\Gamma_{1} }} = p_{L2} } \\ {\frac{{K_{2} }}{{\rho_{2} g}} \cdot \left. {\frac{\partial P}{{\partial N}}} \right|_{{\Gamma_{2} }} = - q_{N} } \\ \end{array} } \right.$$

where *K*_*2*_ is the permeability coefficient of the boundary of the extraction zone, which changes along the direction of workface advancement and vertical direction. *q*_*N*_ is the air leakage per unit area of the boundary of the extraction zone, m^3^/(m^2^·min). *p*_*L2*_ is the simulation area of the working face and the boundary of the extraction zone from the working face inlet *L*^*'*^m at the boundary wind pressure, in Pa. $p_{L2} = L^{\prime}(p^{\prime}_{1} - p^{\prime}_{2} )/L^{\prime}_{0}$, $p^{\prime}_{1}$ is the wind pressure at the inlet of the working face in Pa, $p^{\prime}_{2}$ is the wind pressure at the outlet of the working face in Pa, $L^{\prime}_{0}$ is the length of the simulated working face in m.

The on-site measurement indicates that the air supply to the working face of the 9# coal seam is 1910 m^3^/min, with a pressure difference of 90 Pa between the upper and lower outlets. The length of the working face is 200 m, with an air leakage rate of 8% to the air mining area. In the simulation, the wind speed in the air intake lane is set at 1.5 m/s, the outlet of the return lane is designated as a free flow, the outlet of the gas control lane is a pressure outlet, the temperature of the wind flow and the initial temperature of the mining area is set at 21 °C, and the gravitational acceleration is 9.8 m/s^2^.

### Model building

In order to reduce the complexity and computational complexity of the model, the following assumptions are made: (1). The coal in the goaf is an isotropic porous medium. (2). The flow of mixed gases in goaf follows Darcy’s law, and there is no chemical reaction between different gas components. (3). Assuming the top and bottom plates of the goaf are walls, i.e. ignoring the impact of air leakage on gas transport in the area.

**Fig. 3:**
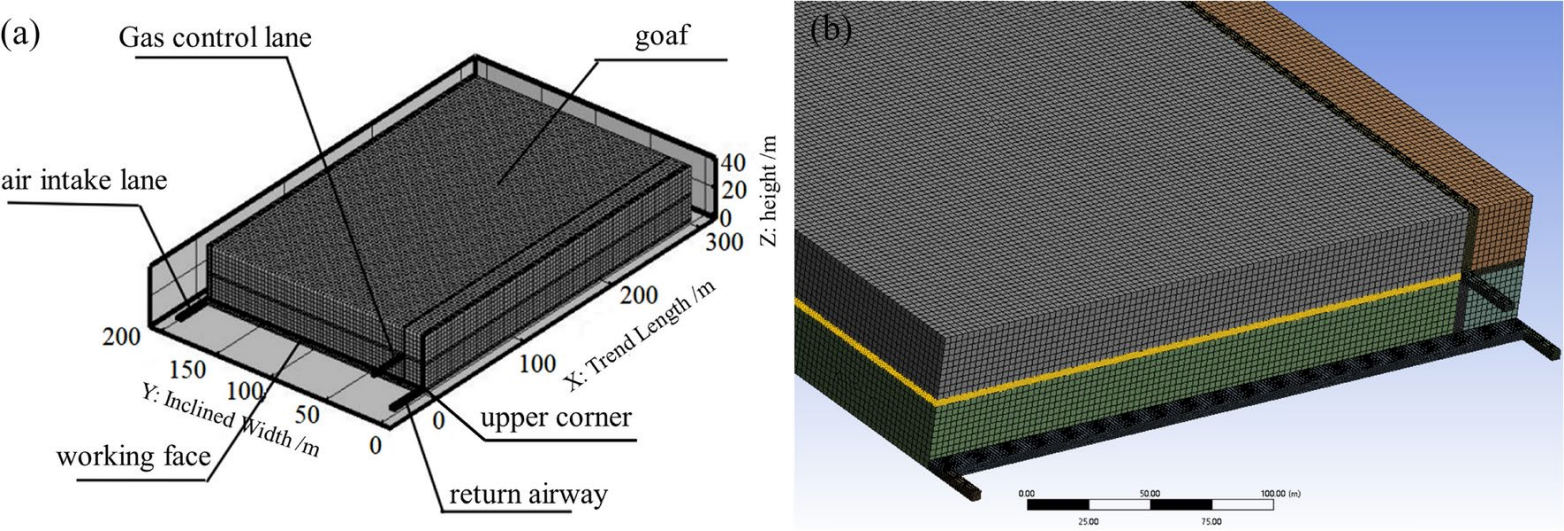
(**a**) Geometric model of goaf. (**b**) Model mesh refinement area.

In accordance with the actual situation on the site of the 9# coal seam in Wuhushan Mine, the geometric model construction and structured grid division were conducted using the SpaceClaim and Meshing software. Additionally, the air inlet section, air return section, high pumping lane, and working face area were locally encrypted in accordance with their respective importance, with the objective of enhancing the accuracy of the calculation. Following the calculation, the number of grid divisions was found to be 511,520. The schematic diagram of the constructed geometric model of the extraction zone and the basic parameters of the geometric model are presented in Fig. 3 and Table 4, respectively.

**Table 4:** Size and basic parameters of the simulated geometric model.

Name	Parameter		
Goaf	Trend Length: 300 m	Inclined Width: 200 m	height: 40 m
Air intake lane	Length: 30 m	Width: 5 m	Height: 3 m
Return airway	Length: 30 m	Width: 5 m	Height: 3 m
Gas control lane	Length: 30 m	Width: 3 m	Height: 3 m
Working face	Length: 200 m	Width: 5 m	Height: 3 m

In the Fluent software interface, the pressure-based solver was selected as the simulation tool for the purposes of this study. Subsequently, the energy equation was initiated, and the RNG k-epsilon model was employed as the viscous model, while the methane-air component model was enabled to accurately simulate the flow characteristics of the gas-air mixture. In establishing the boundary conditions, the velocity inlet boundary was utilized for the air intake lane, with a wind speed of 1.5 m/s. The outlet of the return lane was designated as a free-flow outflow condition, while the outlet of the gas-treatment lane was defined as a pressure outlet boundary, with a maintained temperature of 294.15 K. Furthermore, all wall boundary conditions were set as a no-slip wall, aligning with the actual physical situation.

## Simulation results and analysis

Based on the previous calculation of the height of the fallout zone and fissure zone and the analysis of the reasonable laying level of the gas control lane, the horizontal distances of the gas control lane (from the horizontal distance of the return-air lane of the working face) were determined to be 15, 20, and 30 m, and the vertical distances (from the top of the coal seam) were determined to be 15, 20, 25, and 30 m in simulating and analyzing the effect of the pumping of the gas control lane.

**Fig. 4:**
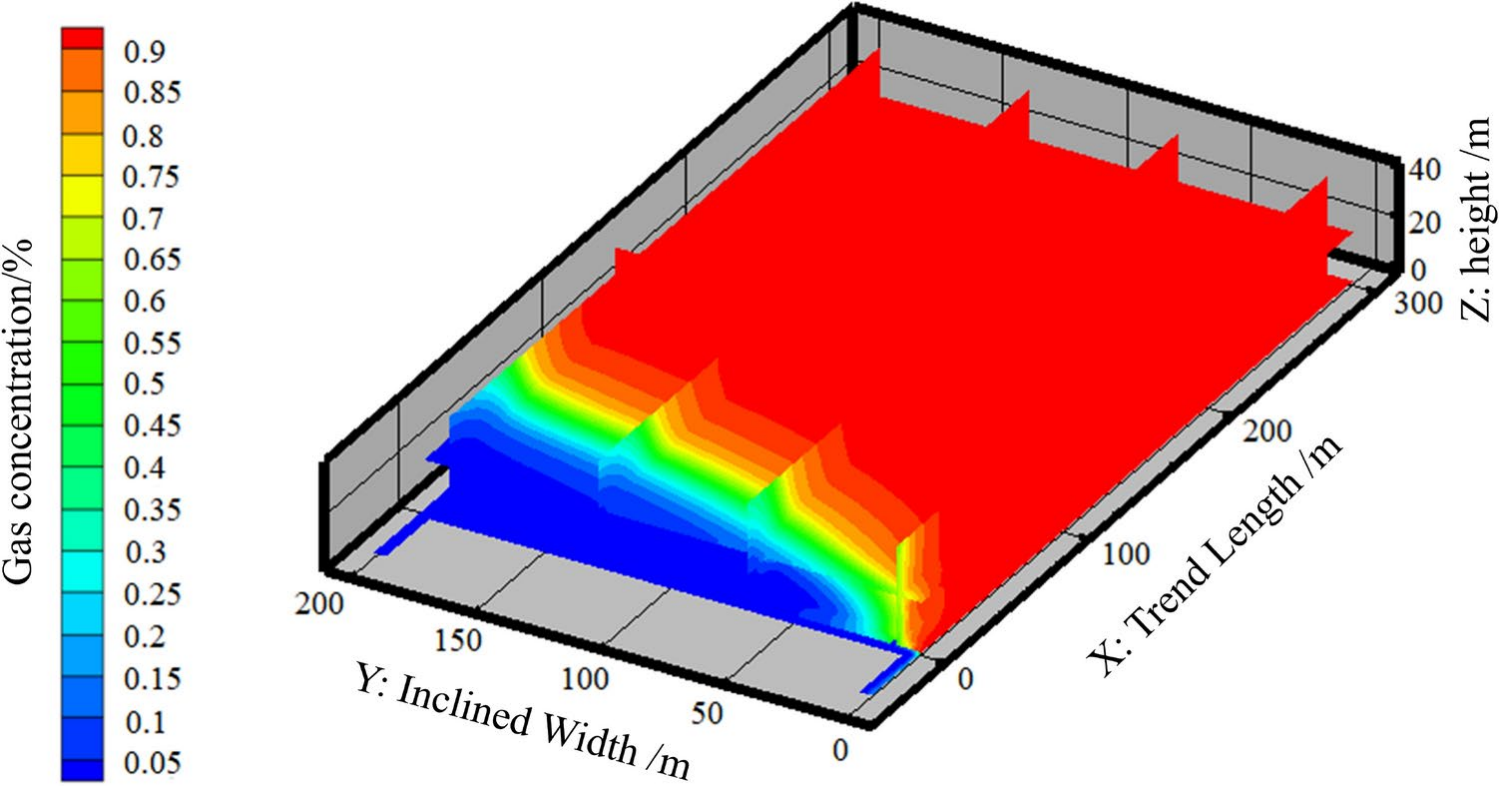
Gas concentration distribution map of goaf without drainage.

In order to be able to more intuitively react to the extraction effect of the gas management lane, first of all, the gas concentration field of the 9# coal seam air-mining area under the condition of no extraction is simulated and analyzed by FLUENT software, and the simulation results are shown in Fig. 4.

As illustrated in Fig. 4, the majority of the gas accumulates in the deeper regions of the extraction zone. This phenomenon can be attributed to the low relative density of the gas, which is subjected to upward buoyancy. This results in the release of gas from the relict coals and the surrounding rocks, typically propagating through the cracks to the conductive fracture zones, where they accumulate. This, in turn, creates a concentration of the gas.

The mean gas concentration at a distance of 50 m from the working face on the return side was found to be 35%, while the concentration at the same distance on the air intake lane was only 0.04%. This phenomenon can be attributed to the leakage of fresh airflow into the air pocket on the air intake lane of the working face, which resulted in the dilution of the gas in the air pocket and its subsequent flow back to the working face from the return air. This led to a high concentration of gas on the return side of the working face, which in turn caused the gas concentration to exceed the permissible limit in the upper corner of the working face.

The horizontal distance has been determined to be 15, 20, or 30 m, while the vertical distance has been determined to be 15, 20, 25, or 30 m. This has resulted in a total of 12 different gas management lane layout schemes, based on the actual situation in the field. For this simulation and analysis, a more representative selection of six cases has been chosen, as detailed in the Table 5.

**Table 5:** Layout horizon parameters of simulated time-high pumping roadway.

name	1	2	3	4	5	6
Horizontal distance L (m)	20	20	20	20	15	30
Vertical distance H (m)	15	20	25	30	20	20

**Fig. 5:**
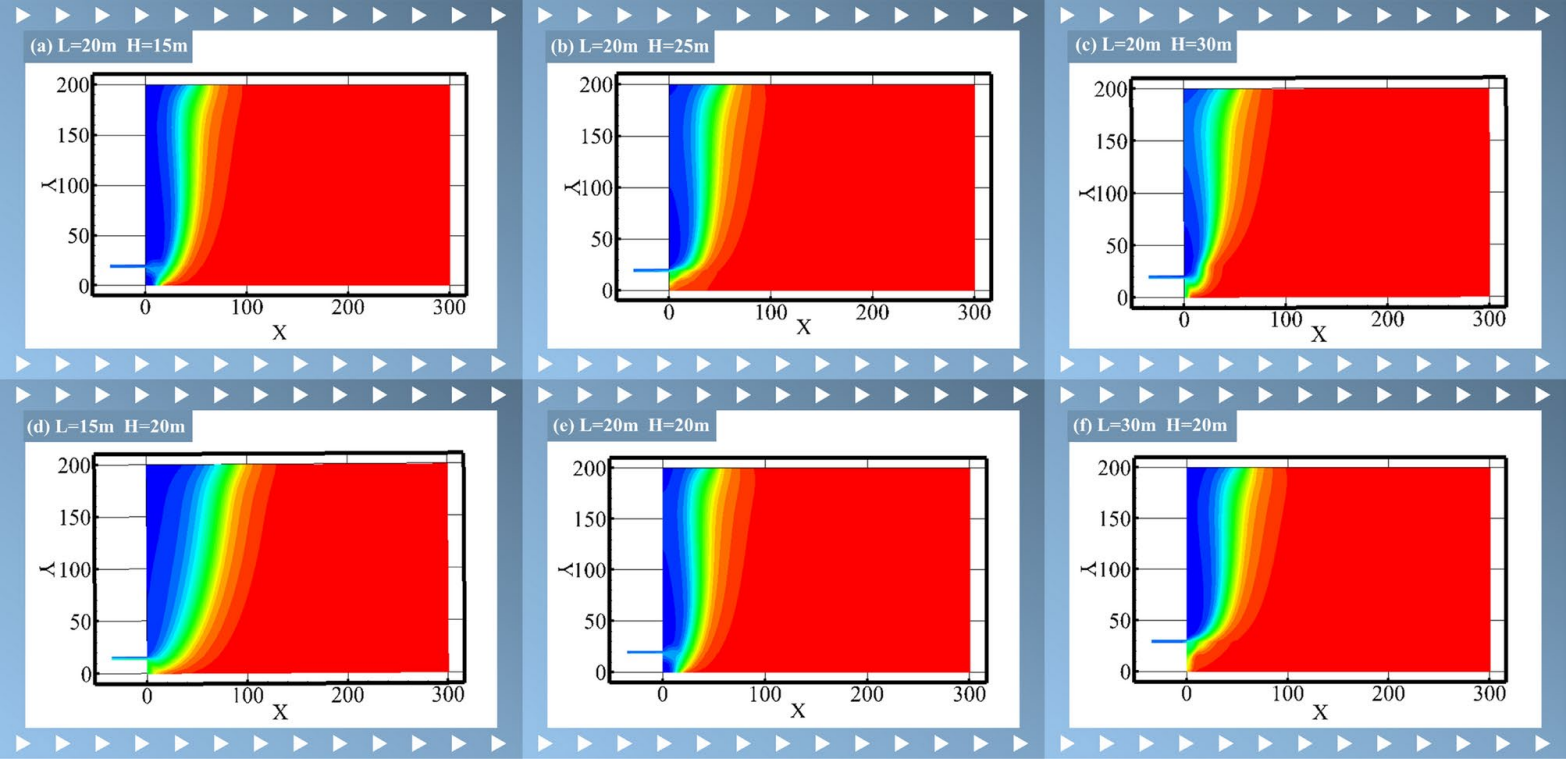
Distribution of gas concentration field in the extraction area of different layers of high extraction lane.

Figure 5 shows the distribution of gas concentration field in goaf when the gas control roadway is located at different positions. From the distribution map of the gas concentration field, it can be seen that when the vertical distance of the gas treatment roadway is the same, as the horizontal distance increases, the gas treatment effect of gas treatment roadway near the upper corner weakens; When the horizontal spacing of the gas control roadway is the same, the gas control effect in the goaf shows significant differences at different levels. At the same time, compared with the nonextraction state in Fig. 4, it can be seen that the installation of gas control tunnels greatly reduces the gas concentration in the upper corner and further expands the low gas area, enhancing the reliability of safe production in the working face.

### The relationship between the layout layer and the gas concentration in the upper corner

**Fig. 6:**
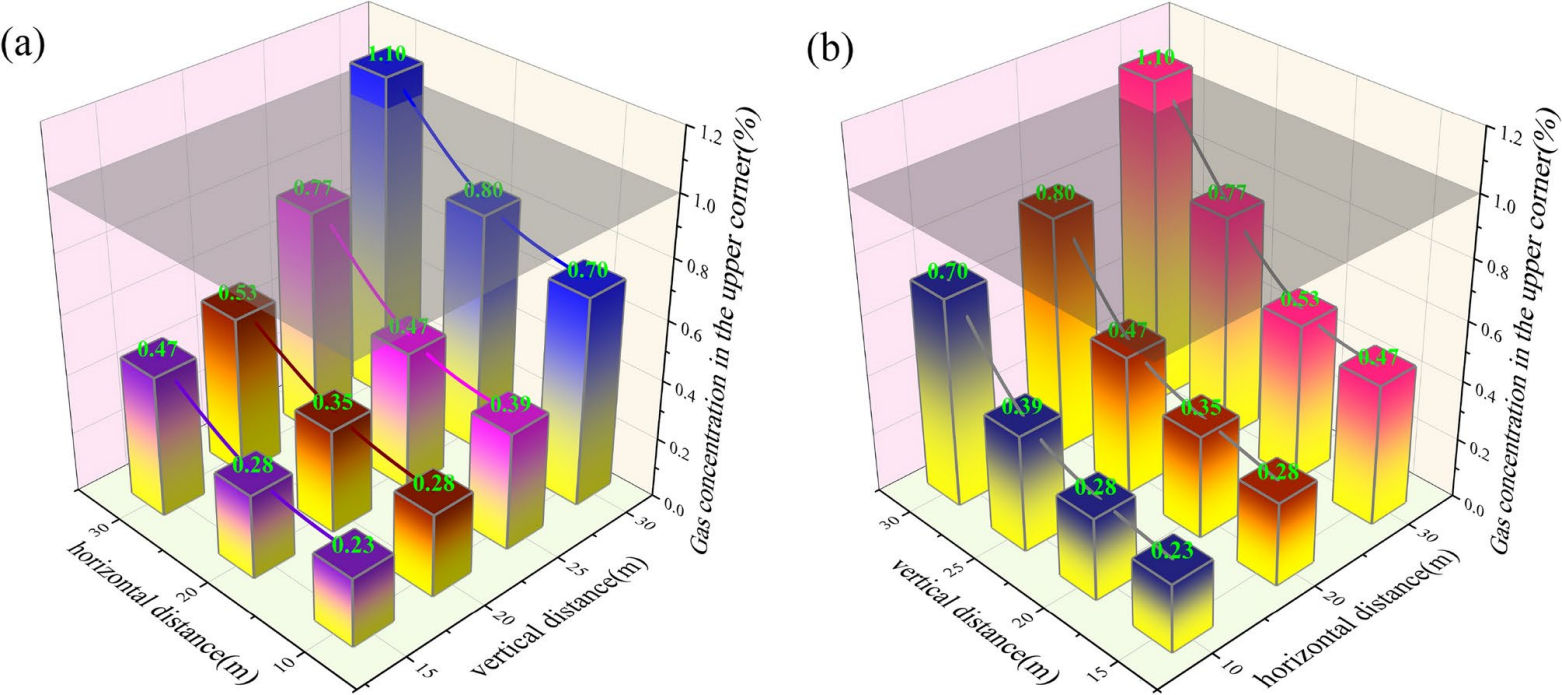
Relationship curve between high roadway layout horizon and gas concentration in the upper corner.

Draw the relationship curve between the gas control roadway layout layer and the gas concentration at the corner of the working face according to Fig. 5, as shown in Fig. 6. From Fig. 6a, it can be seen that when the horizontal distance of the gas control roadway does not exceed 20 m, as the horizontal distance of the gas control roadway increases, the gas concentration in the upper corner slowly increases, and the slope of the function is small at this time. The minimum amplitude of gas change in the upper corner is only 0.05%; With the continuous increase of the horizontal distance of the gas control roadway layout, when the horizontal distance of the gas control roadway layout exceeds 20 m, the gas concentration at the upper corner significantly increases. At this time, the maximum gas concentration increases from 0.8 to 1.11%, with a change amplitude of 0.31%.

The main reason is that when the horizontal distance of the gas control roadway does not exceed 20 m, the gas concentration on the return air side of the goaf is affected by the gas control roadway extraction; When the horizontal distance exceeds 20 m, the impact of the gas control roadway on the gas on the return air side of the goaf weakens, and the degree of correlation with the gas in the central area of the goaf increases. As a result, in the initial stage, with the increase of the horizontal distance of the gas control roadway, the gas concentration in the upper corner remained basically stable, and the growth rate was relatively small; In the later stage of increasing the horizontal distance, the growth rate significantly increased. Using 1% as the upper corner gas concentration exceeding the limit (as shown in the dashed plane in the figure), it can be seen that when the vertical distance is 30 m, only when the horizontal distance does not exceed 27.2 m, it will not cause the occurrence of gas concentration exceeding the limit; When the vertical distance is less than 30 m, arranging gas treatment roadway extraction at any position in the plan can ensure that the gas concentration is below 1% and will not cause gas concentration to exceed the limit. At the same time, regardless of the location of the gas control roadway, an increase in horizontal distance will cause an increase in gas concentration in the upper corner, and the larger the horizontal distance, the more obvious the trend of change, which is consistent with the actual situation on site.

Figure 6b illustrates that the vertical distance of the gas control roadway layout and the gas concentration curve in the upper corner are exponential functions. When the horizontal distance of the gas control roadway layout is 10 m and 20 m, irrespective of the vertical distance within the range of 15–30 m, the gas concentration in the upper corner is below 1%. When the horizontal distance of the gas control roadway is increased to 30 m, it can only meet the requirements of coal mine safety regulations when the vertical distance is not less than 28.5 m. As the vertical distance increases, the gas concentration in the upper corner also increases, which is consistent with the aforementioned characteristics of the gas in the goaf.

### Analysis of the relationship between the layout layer and the gas concentration in the return airway

**Fig. 7:**
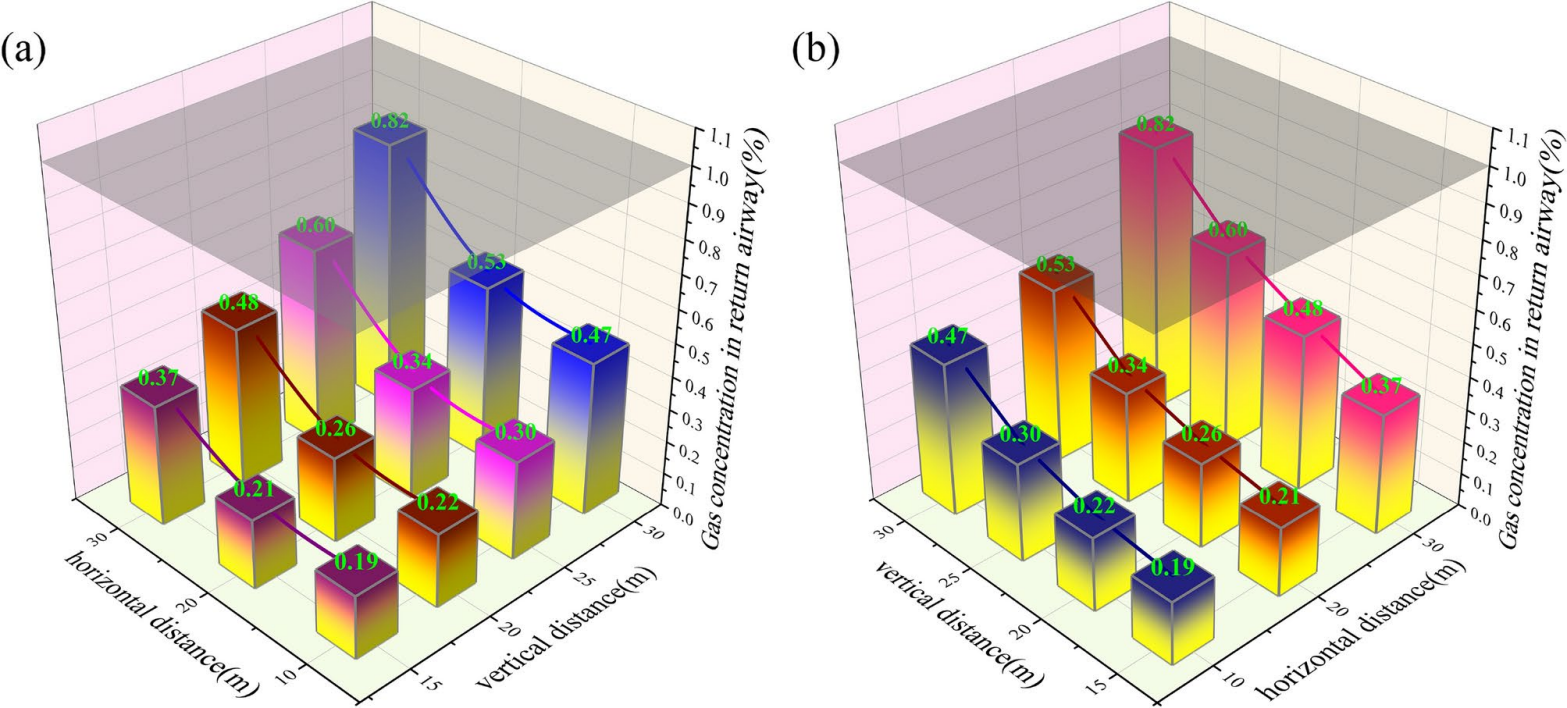
Relationship curve between layout horizon of high drainage roadway and gas concentration in return air roadway.

Figure 7 illustrates the relationship between the gas control roadway layout layer and the gas concentration in the return airway. As illustrated in Fig. 7a, when the horizontal distance of the gas control roadway layout is less than or equal to 20 m, the gas concentration in the return airway gradually increases with the expansion of the horizontal distance of the gas control roadway. The maximum variation in gas concentration within the return airway is 0.03%. However, when the horizontal distance of the gas control roadway layout exceeds 20 m, the gas concentration at the upper corner experiences a notable surge. At this juncture, the maximum gas concentration increases from 0.53 to 0.82%, representing a variation of 0.29%.

As shown in Fig. 7b, with the increase of vertical distance, the gas concentration in the return airway shows an upward trend, and the change trend gradually decreases. When the horizontal distance of the gas control roadway is less than 30 m, regardless of the vertical distance within the range of 15–30 m, the gas concentration in the return airway will not exceed the limit. The results show that as the vertical distance increases, the horizontal distance of the gas control roadway layout weakens the gas control effect on the return airway, and the larger the vertical distance, the worse the gas control effect.

### The relationship between the layout layer and the gas concentration in the roadway

**Fig. 8:**
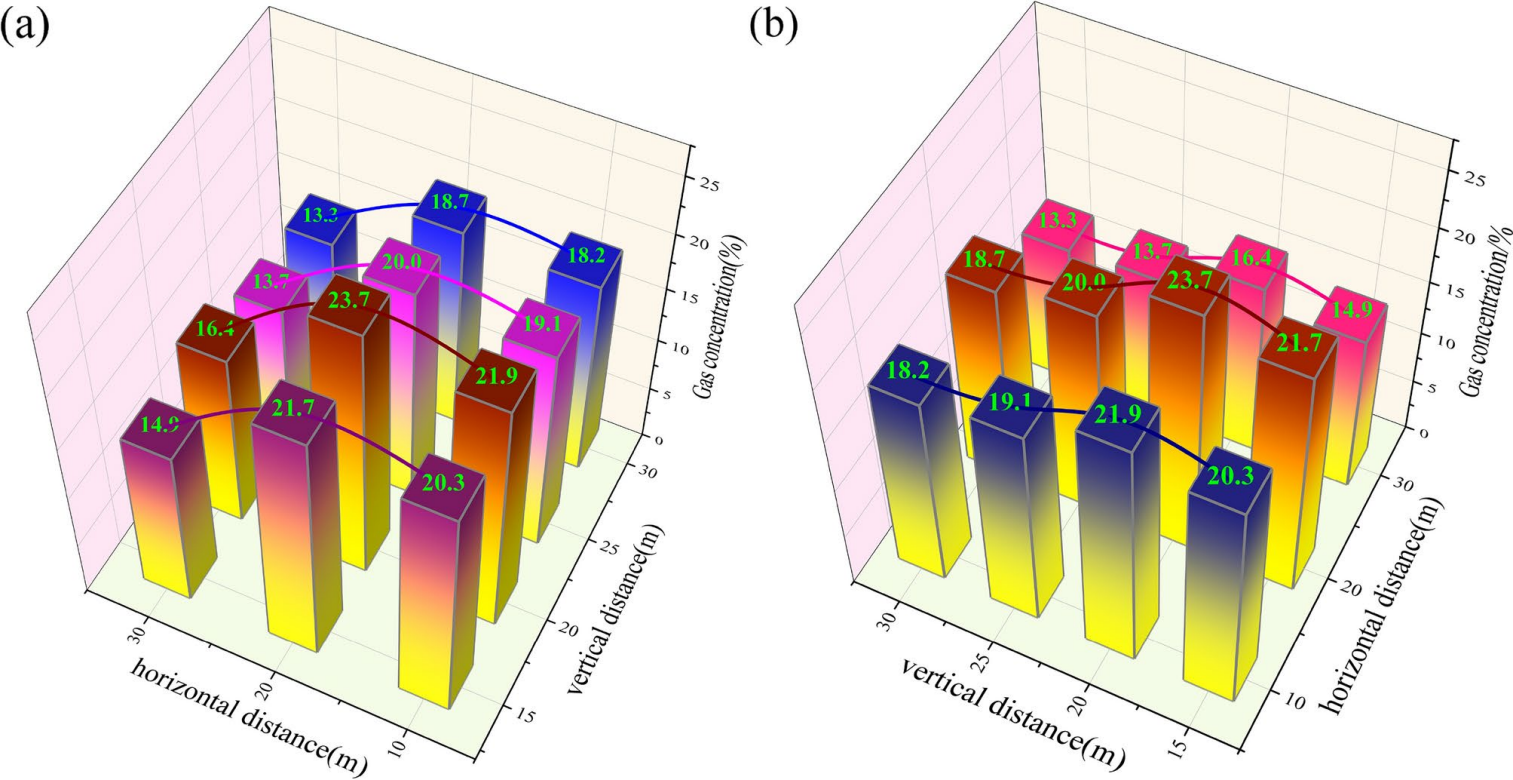
The relationship curve between the level of the high pumping lane and the gas concentration of the high pumping lane.

Figure 8 illustrates the relationship between the configuration of gas control tunnels and the concentration of gas within these structures. Figure 8a illustrates that the relationship curve between horizontal distance and gas extraction concentration in gas control tunnels under different vertical distance conditions is approximately parabolic, exhibiting growth stages, decrease stages, and peak values. This indirectly reflects the significant impact of different horizontal distances on gas extraction efficiency in gas control tunnels. As illustrated in the figure, when the horizontal distance of the gas control roadway is less than 20 m, the increase in the extraction concentration of the gas control roadway is relatively gradual, with a maximum increase of 1.8%. Additionally, the peak concentration of gas extraction in gas treatment tunnels at different vertical distances is observed at a horizontal distance of 20 m, with a maximum value of 23.7% and a minimum of 18.7%. Conversely, when the horizontal distance of the gas control roadway exceeds 20 m, the extraction concentration exhibits a notable decline.

As illustrated in Fig. 8b, the maximum value of the gas extraction concentration in the gas control lane is observed when the laying distance is 20 m. This value is observed for different spacing configurations. When the laying distance exceeds 20 m, the gas extraction concentration of the gas control lane demonstrates a declining trend. With the extension of laying distance, the gas extraction concentration continues to diminish, and the change amplitude gradually diminishes. At a laying distance of 30 m, the gas extraction concentration of all laying locations of the gas control lane attains its lowest value, with a maximum value of 18.7% and a minimum value of 13.3%. The theoretical analysis indicates that the initial increase and subsequent decline in gas extraction concentration can be attributed to the fact that when the gas control lane is laid with a vertical distance of 15 m, only the gas generated by the coal remains can be extracted. Conversely, when the gas control lane is laid with a vertical distance of 20 m, it can be utilized to extract gas from the coal remains of the mining area and effectively intercept the gas outflow from the neighboring layers. As the vertical distance is increased further, the influence of air leakage from the mining area drives the wind flow, which results in a reduction of the extraction concentration of the gas control lane. Secondly, the increase in vertical distance leads to the gas control lane being situated at a considerable distance from the main gathering area of the gas in the mining area, which results in a significant decrease in the extraction concentration.

### Relationship between layer layout and gas extraction purity

**Fig. 9:**
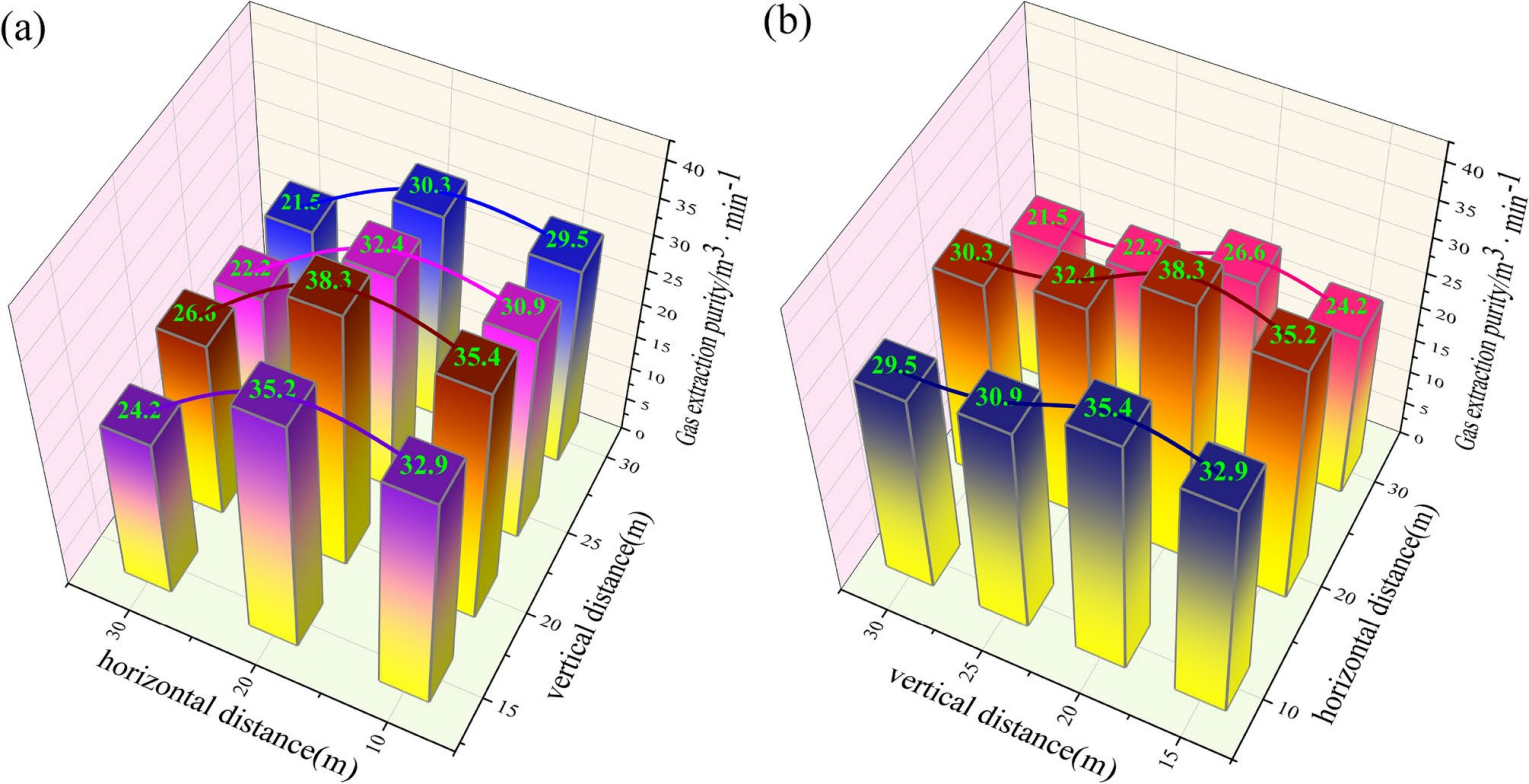
Relationship curve between gas control roadway layout layer and gas extraction purity.

Figure 9 illustrates the correlation between the gas control roadway layout layer and the gas extraction stock. Figure 9a illustrates that the horizontal spacing of gas control tunnels has a notable influence on the volume of pure gas extracted, with a correlation that can be approximated as quadratic. When the horizontal distance of the gas treatment roadway is less than 20 m, the pure gas extraction volume increases gradually with an initially slow-rising speed as the horizontal distance of the gas treatment roadway increases. The maximum can increase from 35.4 m^3^/min to 38.3 m^3^/min. Upon reaching a horizontal distance of 20 m, the gas extraction purity demonstrates a downward trend, with a more pronounced decline. As the horizontal distance continues to increase, the slope of the gas extraction purity curve becomes more pronounced, resulting in a significant change, with a maximum decrease from 38.3 to 26.6 m^3^/min. The arrangement of the gas control roadway at any vertical distance has a significant impact on the gas extraction purity of the goaf. An increase in the horizontal distance results in a notable change in gas extraction purity, with a greater effect observed as the horizontal distance increases.

As shown in Fig. 9b, when the vertical distance of the gas control roadway is less than 20 m, the pure gas extraction volume gradually increases with the increase of the vertical distance of the gas control roadway, and the maximum can increase from 35.2 to 38.3 m^3^/min; When the vertical distance is 20 m, the pure gas extraction reaches its maximum value; When the vertical distance exceeds 20 m, the pure gas extraction decreases significantly with the increase of the vertical distance of the gas control roadway; When the vertical distance exceeds 25 m, the pure gas extraction rate decreases slowly, with the minimum change amplitude being only 0.7 m^3^/min. Therefore, when the vertical distance is less than 20 m, an increase in the vertical distance will lead to a significant increase in the net amount of gas extraction in the goaf, and when the vertical distance is 20 m, the rate of increase in the net amount of gas extraction is the fastest; When the vertical distance is greater than 20 m, the gas extraction purity shows a significant downward trend, and the larger the vertical distance, the slower the downward speed.

In summary, when the gas control alley is laid out with level distance L = 20 m and vertical distance H = 20 m, although the gas concentration in the upper corner and the return airway does not reach the lowest value in all the schemes, but it is closer to the lowest value of 0.23% and 0.19% in different schemes, at the same time, the concentration of the gas extraction in the gas control alley at the same location and the pure volume of the gas extraction are the highest value in different schemes, with the concentration of the gas extraction being 23.7% and the pure volume of the gas extraction being 38.3 m^3^/min. The gas extraction concentration is 23.7%, and the pure extraction volume is 38.3 m^3^/min. Therefore, it is determined that the reasonable laying level of the 9# coal seam gas control lane for gas control in Wuhushan Mine is L = 20 m and H = 20 m. At this time, the gas control lane can give full play to the role of extraction, and a larger range of gas extraction can be carried out, which can effectively solve the problem of over-limit of gas on the upper corner of the working face and efficiently extract the high concentration of gas in the air-sealed area.

## Measured data on site

**Fig. 10:**
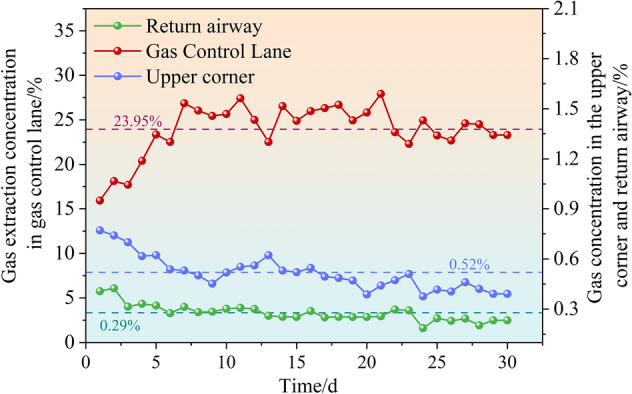
The relationship curve between gas concentration and time is detected at different locations (i.e. upper corner, return airway, gas treatment airway).

The layer position, as determined in the numerical simulation experiment, was used to collate and analyze the gas concentration data in the upper corner of the working face of the 9# coal seam in Wuhushan Mine, as well as the gas concentration data in the return airway in the 30d after the gas control lane can be pumped. The resulting data was plotted as a gas concentration vs. time curve, as shown in Fig. 10.

Figure 10 illustrates that the mean gas concentration in the upper portion of the working face of the 9# coal seam in the Wuhushan Mine is 0.52%, which is 0.17% disparate from the simulation result of 0.35%. The gas concentration in the return airway was 0.29%, a difference of 0.03% from the simulation result of 0.26%. The mean gas extraction concentration in the gas management lane is 23.95%, which is 0.25% different from the simulation result of 23.7%, and the discrepancy in the pure volume of gas extraction in the gas management lane is 1.8 m^3^/min. The on-site situation and the numerical simulation results are in general agreement, which indicates that the numerical simulation process of this thesis is correct, the simulation results are reliable, and the gas management lane set up according to the conclusion of the simulation can effectively manage gas in the working face of the 9# coal seam. The gas control roadway set up based on simulation conclusions can effectively control the gas in the 9# coal seam working face.

## Conclusion

1. Through the in-depth study of the theory of reasonable arrangement of gas control lane, the basic guideline for the location of gas control lane is determined in both horizontal and vertical directions. At the same time, with the help of empirical formulas, it was calculated that the height of the fallout zone in the 9# coal seam of Wuhushan Mine was between 6.86 and 11.26 m, while the height of the fissure zone was within the range of 30.11–41.31 m.
2. This study conducted a systematic theoretical analysis of the porosity of the fissure zone and the fallout zone in the air-mining area, revealing the changing rules and influencing factors of the porosity in these two zones and proposing a corresponding calculation method. Based on the on-site measured data of 9# coal seam working face, the distribution characteristics of the porosity of the fallout rock in the mining area are further determined. The calculation results show that the porosity in the porosity increase area of the fissure zone is 14.6%, while the porosity in the recovery area is 9.3%.
3. Based on the actual conditions of the site, a numerical simulation model of the 9# coal seam in Wuhushan Mine was constructed, and a detailed numerical simulation analysis was carried out on the gas concentration field of the mining area without gas control lanes and with gas control lanes laid in six different layers. The simulation results show that the average gas concentration at the 50 m distance from the working face on the return airway side and the air intake lane are 35% and 0.04%, respectively, in the case of no gas control lane. The problem of gas overlimit in the upper corner of the working face can be effectively solved through the reasonable deployment of a gas control lane.
4. After a comprehensive assessment of six kinds of gas control lanes laid in different layers, including the comparison of four indexes: the gas concentration in the upper corner of the working face, the gas concentration in the return airway, the gas concentration in the gas control lane, and the pure amount of gas extraction, this study establishes the optimal laying scheme for the gas control lane in the 9# coal seam of the Wuhushan Coal Mine: i.e., at a level distance and vertical distance of 20 m (the corresponding index values are 0.35%, 0.26%, 23.7%, 38.3 m^3^/min), the gas control effect is most significant. The on-site verification results show that compared with the monitoring data, the errors of gas concentration in the upper corner, gas concentration in the return airway, gas extraction concentration and extraction purity in the gas treatment airway under this layout plan are 0.17%, 0.03%, 0.25%, and 1.8 m3/min, respectively, which fully confirms the accuracy of the simulation prediction.

## Supplementary Information

Supplementary Information.

## Data Availability

Data is provided within the supplementary information files.
